# Moult-Induced Changes in Gut Microbiota of African Penguins (*Spheniscus demersus*)

**DOI:** 10.3390/ani16030468

**Published:** 2026-02-02

**Authors:** Jingle Jiang, Di Hu, Hongyun Shi, Kangning Huang, Jianqing Zheng, Enle Pei

**Affiliations:** Shanghai Endangered Species Conservation and Research Centre, Shanghai Zoo, Shanghai 200335, China

**Keywords:** African penguins, gut microbiota, moult

## Abstract

The gut microbiota of seabirds can undergo shifts during the moulting period due to fasting. Understanding the characteristics of gut microbiota in African penguins (*Spheniscus demersus*) throughout moulting is meaningful for the conservation and management of this critically endangered seabird species. A total of 12 captive African penguins reared in Shanghai Zoo were divided into three sampling stages: (1) pre-moulting (Pre); (2) mid-moulting (Mid); and (3) post-moulting (Post). The 16S rDNA gene sequencing of faecal samples was performed to evaluate differences in diversity, composition, and predicted function of the gut microbiota across the moulting period. The results showed that Firmicutes, Proteobacteria, and Actinobacteria were the predominant bacterial phyla in African penguins throughout moulting. Moulting significantly altered the diversity and composition of gut microbiota in captive African penguins. Pre-moulting penguins exhibited higher abundances of Bacilli and Lactobacillales compared with mid- and post-moulting groups, whereas mid- and post-moulting penguins had increased abundances of several potentially harmful bacteria. In addition, the functional prediction analysis indicated that pre-moulting penguins had a higher abundance of protein N-glycosylation pathway compared with both mid- and post-moulting penguins. The pre-moulting group also exhibited a decreased abundance of the vitamin E biosynthesis pathway compared with the mid-moulting group. The present study has revealed that the gut environment of African penguins is more vulnerable during the mid- and post-moulting periods. The findings provide reference information for improving the management of endangered seabird species during moult.

## Simple Summary

African penguins (*Spheniscus demersus*) are critically endangered seabirds that go through a stressful annual moulting period. This study investigated how the microbial structure in their gut changes before, during, and after moulting, as these microbes are vital for health and digestion. The results of high-throughput sequencing showed that moulting significantly changed the diversity and composition of gut microbiota. Before moulting, penguins had more beneficial bacteria, but during and after moulting, the abundances of potentially harmful bacteria increased, making their gut environment more vulnerable. The findings suggest that African penguins are at higher health risk in the mid- and post-moulting stages. This information is helpful for improving the ex situ conservation strategies of seabirds by recommending strategies like reducing stress, allowing natural fasting, and considering nutritional support during moult.

## 1. Introduction

The gut microbiota plays a crucial role in regulating host physiological functions, including gut health, the digestion and absorption of nutrients, immunity, and metabolism [1,2,3]. Maintaining a dynamic balance of gut microbiota is important for the survival and environmental adaptation of wild animals [4,5]. The characteristics of gut microbiota in endangered wild animals have attracted considerable research attention [6,7,8,9]. Research on the gut microbiota could provide fundamental information for understanding the physiological traits of endangered species, and it is beneficial for the conservation and management of endangered species.

The wild populations of many seabird species are declining due to human intervention and environmental alterations [10,11,12]. In seabirds, the moulting period represents a particularly vulnerable life stage characterised by intensive physiological stress and extremely enhanced energy demands. The metabolic requirements for feather formation and thermoregulation also dramatically increase during moult [13]. Most seabirds, including penguins, undergo a fasting period during moult. The feed intake and body weight of penguins increase prior to moulting but decline sharply after moult begins. These alterations in feeding behaviour may change the structure of gut microbiota, thereby potentially affecting the health of penguins during moult. In the wild, penguins experience a high mortality rate during and after moult, partly due to insufficient storage of fat and protein [14,15]. There is a possibility that a moult-induced alteration in gut microbiota could lead to gut dysfunction, contributing to high mortality. Current studies have primarily investigated the effects of fasting in Antarctic penguins during moult (early moult vs. late moult) or during feeding and moulting periods [13,15]. A significant increase in bacterial diversity was observed in wild gentoo penguins (*Pygoscelis papua*), whereas no such change was observed for wild chinstrap penguins (*Pygoscelis antarcticus*) between the feeding and moulting periods [13]. Additionally, in wild king penguins (*Aptenodytes patagonicus*), the abundances of both Proteobacteria and Bacteroidetes reduced during moult. In wild little penguins (*Eudyptula minor*), decreased Firmicutes and increased Bacteroidetes abundances were observed from early to late moult, respectively [15]. However, the changes in gut microbiota in penguins after moult have not been elucidated yet. Investigating the dynamics of gut microbiota throughout the moulting cycle is important for understanding the physiological and metabolic characteristics of penguins.

The African penguin (*Spheniscus demersus*) is one of the endangered seabird species which is mainly distributed along the coast of South Africa [16,17]. Over the past 30 years, the wild breeding population of African penguins has declined by 77.8% [18]. Recently, this species has been listed as critically endangered [19,20]. Little is known regarding the internal physiological changes in African penguins during moult. In the wild, penguins might hunt different fishes before and after moult, which might interfere with the results of the microbiome. In contrast, captive penguins are maintained in a controlled environment with a routine diet. This offers a suitable model for investigating moult-induced changes in the gut microbiota of penguins.

Thus, the present study used captive African penguins to conduct the first characterisation of gut microbiota dynamics across pre-, mid-, and post-moulting stages in seabirds. The results could also provide a theoretical basis for understanding the physiological characteristics of seabirds and optimising ex situ conservation strategies during the moulting period.

## 2. Materials and Methods

### 2.1. Animals and Sample Collection

**Figure 1:**
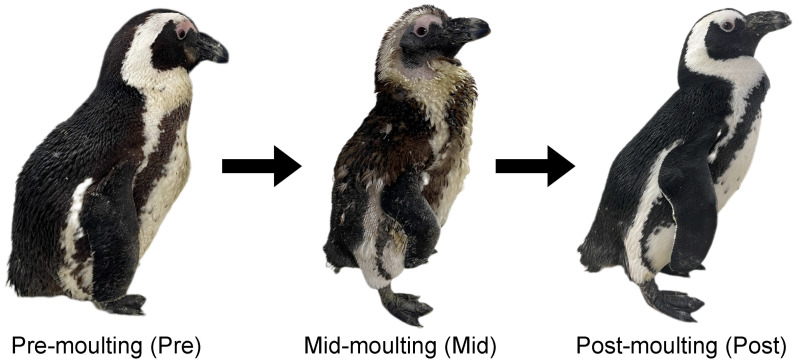
Different moulting stages of African penguins. Pre-moulting: penguin with significantly increased weight gain and loose plumage. Mid-moulting: penguin that has shed approximately half of its plumage. Post-moulting: penguin that has replaced all feathers and resumes swimming behaviour.

A total of 12 captive African penguins were included in this study. All penguins involved in this study were healthy, which was determined by timely blood assay, captive-bred, and reared in the same environment in Shanghai Zoo. Each individual moulted once a year regularly after the breeding season. The individual information of age, sex, and moulting stage is shown in Table 1. Moulting stages were distinguished based on body weight and feather condition (Figure 1). The pre-moulting phase was defined by significantly increased weight gain and loose plumage. The mid-moulting phase was identified when approximately half of the plumage had been shed. The post-moulting phase was characterised by complete feather replacement and resumed the swimming behaviour.

The sampling stage was divided into pre-moulting (Pre), mid-moulting (Mid), and post-moulting (Post). Faecal samples were collected indoors from sterilised floors. Immediately after defecation, the dark and solid portions of the guano were collected using sterile forceps at different stages. Each set of forceps was used only once and was subsequently disinfected with 70% ethanol to prevent cross-contamination. A total of 36 faecal samples were collected and stored at −80 °C in this study. This study was approved by the Ethics and Animal Welfare Committee of Shanghai Zoo (No. SZ220301).

### 2.2. DNA Extraction and 16S Ribosomal RNA (rRNA) Gene Sequencing

The experimental procedure was performed as previously described [21]. Generally, a QIAampDNA Stool Mini Kit (Qiagen, Redwood City, CA, USA) was applied to extract the total genome DNA. After the determination of DNA concentration and quality, qualified DNA was diluted with sterilised water.

PCR was performed using a Q5^®^ High-Fidelity DNA Polymerase (New England Biolabs, Beijing, China). V3–V4 region of 16S rRNA gene was then amplified, sequenced, and purified. A TruSeq Nano DNA LT Library Prep Kit (Illumina, San Diego, CA, USA) was used to generate the sequencing libraries. After the quality control and quantification, the libraries were sequenced on an Illumina Miseq platform (Illumina, San Diego, CA, USA).

### 2.3. Data Analysis of the 16 S rRNA Gene Sequencing

Quantitative Insights Into Microbial Ecology 2 (QIIME2, v2022.8) was applied to analyse the raw data. Alpha and beta diversity of gut microbiota were analysed in QIIME2 using “ggplot2” (v3.4.4) and “vegan” (v2.6-4) packages, respectively. A linear discriminant analysis effect size (LEfSe) analysis was conducted to analyse the differential biomarkers among all groups. In addition, the sequencing data were further used for functional prediction compared with the Metabolic Pathways From all Domains of Life (MetaCyc) database through the Phylogenetic Investigation of Communities by Reconstruction of Unobserved States 2 (PICRUSt2) software (v2.5.2).

### 2.4. Statistical Analysis

The Kruskal–Wallis test was applied for detecting the significant differences in alpha diversity. Beta diversity was visualised by using plotting principal coordinate analysis (PCoA) based on Bray–Curtis distance. Anosim (analysis of similarities) was applied for the statistical analysis of beta diversity in QIIME2. LEfSe analysis was performed in R software (v4.1.2). The significant threshold for linear discriminant analysis (LDA) score was set at 3.0. The Mann–Whitney test was applied to analyse the data of functional prediction between the groups (Pre vs. Mid, Pre vs. Post, Mid vs. Post). Differences were statistically significant at *p* value < 0.05.

## 3. Results

### 3.1. Microbial Composition in African Penguins Throughout the Moulting Period

**Figure 2:**
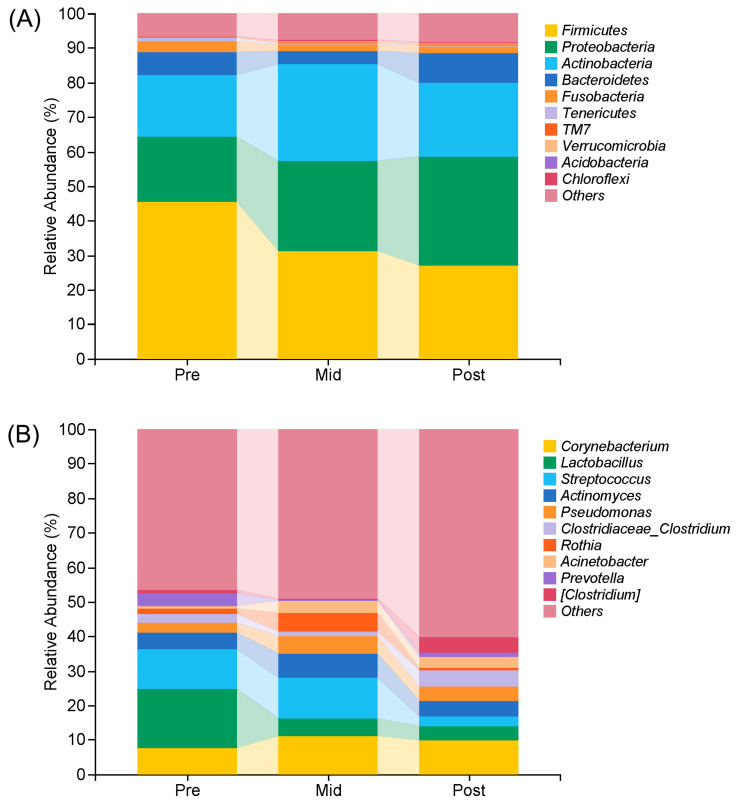
Microbial compositions at phylum (**A**) and genus (**B**) levels in African penguins during moult. Pre, penguin in the pre-moulting period (*n* = 12); Mid, penguin in the mid-moulting period (*n* = 12); Post, penguin in the post-moulting period (*n* = 12). Firmicutes (current name: Bacillota Gibbons and Murray 2021), Proteobacteria (current name: Pseudomonadota Garrity et al. 2021), Actinobacteria (current name: Actinomycetota Goodfellow 2021), Bacteroidetes (current name: Bacteroidota Krieg et al. 2021), Fusobacteria (current name: Fusobacteriota Garrity and Holt 2021), Tenericutes (current name: Mycoplasmatota Murray 2021), TM7 (current name: Minisyncoccota Nakajima et al. 2025), Verrucomicrobia (current name: Verrucomicrobiota Hedlund 2021), Acidobacteria (current name: Acidobacteriota Thrash and Coates 2021), Chloroflexi (current name: Chloroflexota Garrity and Holt 2021).

The top 10 microbial composition at phylum and genus levels in African penguins throughout the moulting period are showed in Figure 2. At the phylum level (Figure 2A), the gut microbiota was dominated by Firmicutes (current name: Bacillota Gibbons and Murray 2021), Proteobacteria (current name: Pseudomonadota Garrity et al. 2021), and Actinobacteria (current name: Actinomycetota Goodfellow 2021). The relative abundance of Firmicutes gradually declined during the moulting period (Pre: 45.65%, Mid: 31.12%, Post: 27.05%). In contrast, the abundance of Proteobacteria increased during moult (Pre: 18.54%, Mid: 26.18%, Post: 31.59%). The relative abundance of Actinobacteria increased at first during the mid-moulting period and then decreased after moult (Pre: 17.93%, Mid: 27.90%, Post: 21.35%).

At the genus level (Figure 2B), *Corynebacterium*, *Lactobacillus*, and *Streptococcus* were the dominant genera in African penguins during moult. *Corynebacterium* showed an initial increase by the mid-moulting period, followed by a decrease after moult (Pre: 7.60%, Mid: 11.09%, Post: 9.87%). *Lactobacillus* was most abundant in the Pre group and gradually decreased during moult (Pre: 17.04%, Mid: 5.23%, Post: 4.13%). *Streptococcus* exhibited similar abundances in the Pre and Mid groups, but its abundance decreased markedly after moult (Pre: 11.54%, Mid: 11.58%, Post: 2.91%). Moreover, most genera were unclassified. The relative abundances of other and unclassified genera gradually increased during moult (Pre: 46.64%, Mid: 48.96%, Post: 60.19%).

### 3.2. Alpha and Beta Diversity Indices of Gut Microbiota in African Penguins Throughout the Moulting Period

**Figure 3:**
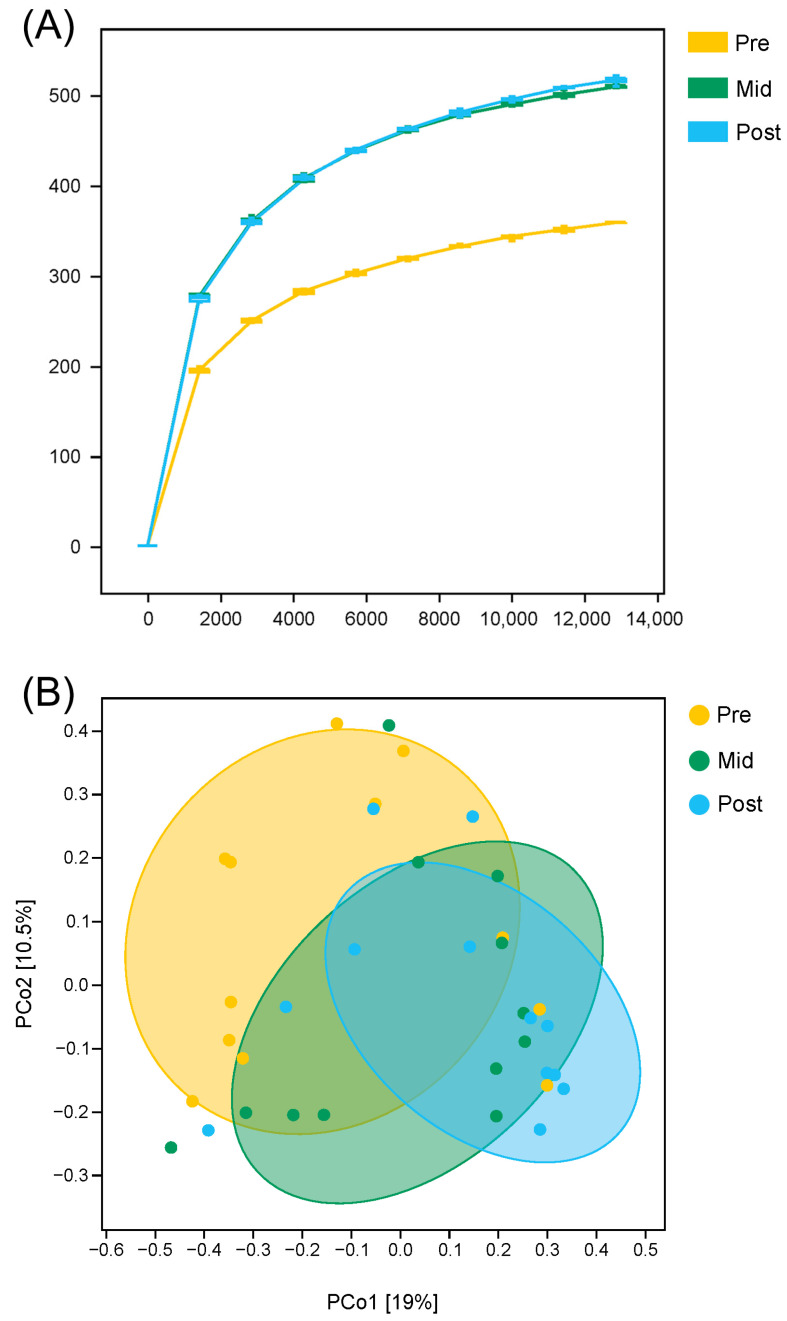
Rarefaction analysis (**A**) and principal coordinate analysis plots (**B**) of gut microbiota in African penguins during moult. Pre, penguin in the pre-moulting period (*n* = 12); Mid, penguin in the mid-moulting period (*n* = 12); Post, penguin in the post-moulting period (*n* = 12).

A rarefaction curve was generated based on the data of the observed species (Figure 3A). The rarefaction curves reached saturation, which indicated that the sequencing depth was large enough for evaluating the diversity of gut microbiota in African penguins throughout the moulting period. The alpha diversity indices are shown in Table 2. The results showed that the Shannon index was altered in all groups. The Shannon index could indicate the diversity of gut microbiota. Both the Mid and Post groups exhibited a significantly higher Shannon index compared with the Pre group (*p* < 0.05), indicating that moult could increase the diversity of gut microbiota. In addition, the results of PCoA (based on Bray–Curtis distance) showed that there was a separation among the groups (Figure 3B). The data of Anosim further indicated that the beta diversity significantly changed between the Pre and Post groups (*p* < 0.05, Table 3). Both alpha and beta diversity indices suggested that moult could change the diversity of gut microbiota in African penguins.

### 3.3. Abundances of Gut Microbiota in African Penguins Throughout the Moulting Period

**Figure 4:**
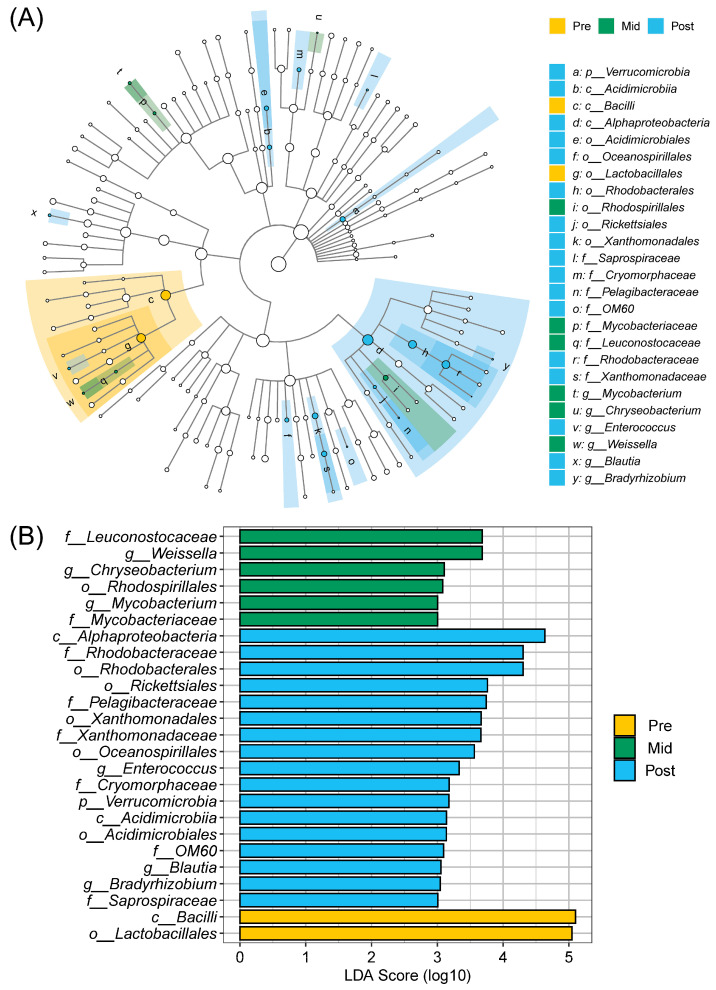
LEfSe analysis of the gut microbiota in African penguins during moult. (**A**) Cladogram plot of biomarkers for all groups. (**B**) Comparison of all groups. Pre, penguin in the pre-moulting period (*n* = 12); Mid, penguin in the mid-moulting period (*n* = 12); Post, penguin in the post-moulting period (*n* = 12). LDA score higher than 3.0 was used as a threshold for significant difference.

The abundance of bacterial taxa was notably different among all groups according to the data from the LEfSe analysis (Figure 4). The Pre group showed increased abundances of Bacilli and Lactobacillales compared to the Mid and Post groups. In the mid-moulting period of African penguins, the abundances of Leuconostocaceae, *Weissella*, *Chryseobacterium*, Rhodospirillales, *Mycobacterium*, and Mycobacteriaceae were higher than those in the pre-moulting and post-moulting periods. Furthermore, the Post group exhibited elevated abundances of Alphaproteobacteria, Rhodobacteraceae, Rhodobacterales, Rickettsiales, Pelagibacteraceae, Xanthomonadales, Xanthomonadaceae, Oceanospirillales, *Enterococcus*, Cryomorphaceae, Verrucomicrobia, Acidimicrobiia, Acidimicrobiales, OM60, *Blautia*, and *Bradyrhizobium* compared to the Pre and Mid groups.

### 3.4. Functional Predictions of Gut Microbiota in African Penguins Throughout the Moulting Period

The putative microbial functions were predicted using the MetaCyc database through the PICRUSt2 software (v2.5.2, Table 4). The Pre group showed higher protein N-glycosylation and lower vitamin E biosynthesis abundances compared to the Mid group (Pre vs. Mid, *p* < 0.05). The Pre group also showed increased protein N-glycosylation abundance and decreased abundances of L-valine degradation, aerobactin biosynthesis, mevalonate pathway, and beta-alanine biosynthesis compared to the Post group (Pre vs. Post, *p* < 0.05). In addition, the Mid group exhibited lower abundances of 3-hydroxypropanoate/4-hydroxybutanate cycle, 3-hydroxypropanoate cycle, and heparin degradation compared to the Post group (Mid vs. Post, *p* < 0.05).

## 4. Discussion

The current study provides new insights into moult-induced changes in the gut microbiota of African penguins using 16S rRNA sequencing. The composition of gut microbiota in birds varies with physiological conditions or seasonal changes [22,23,24]. Our previous study has shown that the predominant bacteria of captive African penguins are Proteobacteria (41%), Actinobacteria (25%), and Firmicutes (23%) during the non-breeding season [25], whereas the predominant bacteria are Firmicutes (55%), Proteobacteria (34%), and Actinobacteria (4%) during the breeding season [21]. Herein, we found that, during moult, the dominant phyla of captive African penguins were Firmicutes (35%), Proteobacteria (25%), and Actinobacteria (22%), indicating that the microbial composition differs across the moulting, breeding, and non-breeding periods. Our results have suggested that African penguins have a unique microbial adaptation strategy during physiological transitions. Whether these changes are associated with the season requires further investigation.

Among the three dominant phyla in African penguins throughout the moulting period, Firmicutes was the most abundant bacteria in the gut. Firmicutes can regulate genes involved in the control of fat storage [26,27,28], which is essential for pre-moulting penguins to reserve large amounts of fat. In the progress of moulting, both body weight and Firmicutes abundance decrease continuously. In little penguins, a reduction in the abundance of Firmicutes has also been observed from early to late moult [15]. In addition, Proteobacteria is the most unstable and largest phylum of bacteria, and it is responsive to environmental and dietary changes [29]. The elevated abundance of Proteobacteria reflects an unstable microbial community, and this elevation can usually be observed in gut inflammation or metabolic disturbance [30,31,32]. The current study showed that the abundance of Proteobacteria increased during and after moult. On the contrary, the abundance of Proteobacteria tends to reduce in wild Antarctic penguins during moult [13,15]. It has been demonstrated that captive and wild penguins have distinct microbial structures [33,34]. Variations in the environment might contribute to the differences. Moreover, Actinobacteria are Gram-positive bacteria, which are key for gut homeostasis. Many members of Actinobacteria have been demonstrated to produce antibiotics [35], and they could exert functions of probiotics to enhance the immune system and protect the gut barrier under pathological circumstances [36]. The abundance of Actinobacteria has been shown to reach the highest point in the mid-moulting period (increasing from 18% before moult to 28% in the mid-moulting period). This phenomenon, along with increased Proteobacteria abundance, suggests that the gut microbiota of African penguins might be the most vulnerable during mid-moulting. Therefore, maintaining a stable living environment and minimising the possible stress is particularly important for the ex situ conservation of African penguins during mid-moulting.

A previous study has indicated that microbial diversity was notably elevated in wild gentoo penguins during moult [13]. In agreement, we also found that moulting significantly increased the diversity of gut microbiota in African penguins. Several studies on other animals (mice, hamsters, toads) have shown that fasting could lead to the enhanced diversity of gut microbiota [37,38]. In agreement, increased microbial diversity in this study might be attributed to significantly declined feed intake during moult. The limited nutritional supply can promote competition among various bacteria to increase diversity. Increased microbial diversity might alter the structure of gut microbiota, which potentially causes more colonisation of harmful bacteria. Our results have shown that moult might affect the homeostasis of gut microbiota in African penguins.

The LEfSe analysis has revealed significant differences in the gut microbiota of African penguins throughout the moulting period. A significantly higher abundance of Lactobacillales has been observed in the pre-moulting period compared to the other moulting periods. Lactobacillales belongs to lactic acid bacteria, and it is widely considered as a probiotic, which promotes gut health [39]. Lactic acid bacteria have also been demonstrated to exert anti-inflammatory, antioxidant, and immunoregulatory activities [40,41]. Higher Lactobacillales abundance can also promote digestive function and is beneficial for preventing gut inflammation and other gut diseases [42]. The decrease in Lactobacillales abundance in both mid-moulting and post-moulting periods might represent reduced motility and secretory function of the digestive tract. This could affect the digestive and absorptive functions of African penguins, which might increase the possibility of gut dysfunction and intestinal obstruction during moult. In the wild, penguins lose the water resistance of their feathers during moult, rendering them unable to swim and hunt for fish. In captivity, however, some individuals may still accept food in the mid-moulting period due to the easy availability of fish. If the keepers continue to feed these penguins normally, it may increase the risk of gastrointestinal disorders during moult. Thus, forced feeding should be avoided in the captive management of African penguins during moult. The application of a controlled fasting protocol might reduce the morbidity of gut disorders during moult.

Mid-moulting African penguins had increased abundance of *Chryseobacterium* and *Mycobacterium* in this study. *Chryseobacterium* belongs to an opportunistic pathogen. Even though *Chryseobacterium* has relatively low toxicity, it can induce bacteremia under the circumstances of immune dysfunction [43,44]. *Mycobacterium* is also pathogenic. It has been shown that infection with *Mycobacterium* can cause chronic diseases in penguins, which could eventually develop into granulomatous pneumonia and enteritis [45]. Severe infection with *Mycobacterium* can even lead to death in African penguins [46]. Furthermore, the abundances of other opportunistic pathogens (Rickettsiales, Oceanospirillales and *Enterococcus*) increased in the gut microbiota of post-moulting penguins. A previous study has found that the *Lactobacillus* abundance of laying hens decreases, whereas the abundance of pathogenic bacteria such as *Escherichia coli* markedly increases after moult. Post-moulting hens are more susceptible to Salmonella enteritis [47]. These findings highlight that both mid- and post-moulting periods are critical windows of gut vulnerability, characterised by low probiotic and high pathogenic bacterial abundances. Maintaining gut health should therefore be a priority in the ex situ conservation of African penguins throughout moulting.

To further emphasise the significance of microbial changes, the PICRUSt2 analysis was used to predict moult-related alterations in gut microbiota. During the moulting period, birds’ vitamin consumptions are significantly elevated. A previous study has found that large quantities of vitamin A and E stored in the liver of laying hens are released during moult [48]. Before moult, penguins will eat plenty of fish to reserve the energy and nutrients for moult. Vitamins are mainly derived from the fish. Herein, the function level of vitamin E biosynthesis was significantly enhanced in the mid-moulting period. This result suggested that most of the exogenous vitamins might be depleted in the mid-moulting period. With the start of moult, penguins might need the synthesis of endogenous vitamins to meet their nutritional needs. In addition, supplementation with vitamin E can enhance the immune response of male broiler breeders after moulting [49]. Hence, supplementation with vitamins might recover the nutritional balance and increase the immune response of post-moulting penguins. Additionally, moult could change the function level of protein N-glycosylation in penguins. Protein glycosylation is involved in the regulation of biological processes, including cell signal transduction, protein folding, protein trafficking, and cell recognition [50]. However, the regulatory mechanism of protein N-glycosylation during the moult of seabirds requires future investigation. Our results could establish a foundation for developing microbiota-targeted management strategies to improve the survival outcomes in both captive and wild penguins during moult. The gut of African penguins has been shown to be vulnerable during the mid- and post-moulting periods. According to our results, the following suggestions might improve the management of captive African penguins: (1) environmental stabilisation to minimise stress responses during moult; (2) application of controlled fasting protocols during moult; (3) micronutrient supplementation targeting potential vitamin deficits after moult; (4) probiotics interventions to restore gut microbial balance after moult; and (5) gradual dietary reintroduction protocols after moult. Future research should explore the application prospect of microbial transplantation for captive management and develop non-invasive monitoring tools for wild population conservation.

One limitation of the present study is that the results may not fully reflect the microbial changes during the moult of wild African penguins, who experience variable food availability, climate fluctuations, and predation risk. Future investigations are required to illustrate the microbial differences between wild and captive populations. Additionally, the results of functional prediction based on 16S rRNA sequencing are putative since the functional shifts can only be predicted inferentially based on known genomes. The further application of shotgun metagenomics can reveal the definitive functional changes. Many unclassified genera can also be identified with metagenomics.

## 5. Conclusions

This study demonstrated that moulting significantly changed the gut microbiota of African penguins. Moulting markedly altered the diversity and composition of gut microbiota in captive African penguins. A reduction in beneficial bacterial abundance was observed, with a notable elevation in several potentially harmful taxa at the mid- and post-moulting stages. The putative functional predictions showed that the abundances of protein N-glycosylation decreased and vitamin E biosynthesis pathways increased during moult, respectively. These findings reveal the physiological vulnerability of African penguins during moult and highlight the need for targeted management strategies (such as stress reduction, controlled fasting, and post-moult nutritional support) in ex situ conservation programs.

## Figures and Tables

**Table 1:** Individual information of captive African penguins.

Individual	Age	Sex	Moulting Period
68#	13	Female	14–29 October 2023
94#	11	Female	30 October–11 November 2023
110#	11	Female	5–24 November 2023
113#	11	Female	29 September–14 October 2023
114#	11	Male	17 November–29 November 2023
120#	10	Female	7–24 November 2023
122#	10	Female	30 October–16 November 2023
130#	9	Male	17 November–2 December 2023
134#	8	Male	12–31 January 2024
146#	5	Female	24 November–10 December 2023
802#	9	Male	4–17 November 2023
803#	12	Male	16 October–4 November 2023

**Table 2:** Alpha diversity of gut microbiota in captive African penguins during the moulting period.

Alpha Diversity	Pre	Mid	Post	*p* Values
Chao1	403.9	558.8	577.2	0.113
Observed_species	359.1	509.9	517.6	0.071
Goods_coverage	0.995	0.995	0.994	0.648
Pielou_e	0.673	0.733	0.738	0.269
Shannon	5.456 b	6.535 a	6.584 a	0.025
Simpson	0.914	0.953	0.955	0.056

Means with different letters (a and b) within a row are significantly different (*p* < 0.05).

**Table 3:** Beta diversity analysis of gut microbiota in captive African penguins during the moulting period.

Group 1	Group 2	R-Values	*p* Values
All	-	0.078	0.031
Pre	Mid	0.087	0.094
Pre	Post	0.156	0.022
Mid	Post	0.003	0.360

**Table 4:** Functional changes in the gut microbiota of captive African penguins during the moulting period.

Pathway	Description	LogFC	*p* Values
**Pre vs. Mid**			
PWY-7031	protein N-glycosylation	1.433	0.006
PWY-1422	vitamin E biosynthesis	−2.156	0.018
**Mid vs. Post**			
PWY-5789	3-hydroxypropanoate/ 4-hydroxybutanate cycle	−0.665	0.002
PWY-5743	3-hydroxypropanoate cycle	−1.813	0.034
PWY-7644	heparin degradation	−1.631	0.037
**Pre vs. Post**			
PWY-7031	protein N-glycosylation	1.706	0.017
VALDEG-PWY	L-valine degradation	−1.021	0.019
AEROBACTINSYN-PWY	aerobactin biosynthesis	−1.968	0.035
PWY-6174	mevalonate pathway	−1.454	0.037
PWY-3941	beta-alanine biosynthesis	−1.005	0.037

## Data Availability

The original data presented in the study are openly available in Zenodo at https://doi.org/10.5281/zenodo.18158337.
